# Direct input variables: rethinking the limits of the extended mind hypothesis within predictive processing

**DOI:** 10.3389/fpsyg.2026.1860291

**Published:** 2026-10-06

**Authors:** Catalin Teoharie

**Affiliations:** Faculty of Philosophy, University of Bucharest, Bucharest, Romania

**Keywords:** active inference, extended mind, free energy, Markov blanket, predictive processing

## Abstract

Predictive Processing theories and the Free Energy Principle (FEP) face a dilemma when addressing the problem raised by the Extended Mind Hypothesis (EMH): on the one hand, few can deny that some objects, such as phones, computers, or other people, have a disproportionate role in the reduction of free energy; hence, it is natural to accept that the mind is extended. On the other hand, this seems to go against the carefully built framework in which Markov blankets (MBs) create an evidentiary boundary between the brain and the external environment, thus rejecting the claim that the mind can be extended to external objects. One approach would be to allow for permeable MBs, where boundaries can move, hence allowing external objects to become part of the mind. I find these solutions to be unsatisfactory because they go against the FEP framework, and I propose a different solution instead: human agents have the possibility of using Direct Input Variables (DIVs), which involves directly using the results of other models without performing the statistical inference themselves. Reducing free energy can be achieved either by statistical inference performed by the brain or by using DIVs. The use of DIVs removes the need to accept the EMH, preserves the integrity of the FEP framework, and opens new questions regarding how DIVs can be integrated within the FEP framework.

## 1 Introduction

In the wider debate between internalism and externalism, the defenders of the EMH are supporting an active externalism, the more controversial claim that some objects are actually part of cognition and mind, under certain circumstances (Clark, 2008; Kirchhoff and Kiverstein, 2019; Wheeler, 2010). Under these special circumstances, the cognitive processes are being realized by external objects (Clark and Chalmers, 1998) or bodily functions, outside of our brain (Heersmink, 2014; Menary, 2007; Wheeler, 2015). This view is opposing various sorts of internalism, under which brain and nervous system are sufficient for defining what constitutes mind and cognition (Adams and Aizawa, 2010; Hohwy, 2016).

In parallel with discussions around EMH, the predictive processing theories (Friston, 2012; Friston and Kiebel, 2009; Hohwy, 2020; Pezzulo et al., 2021), the Bayesian brain hypothesis (Knill and Pouget, 2004) and the FEP (Friston, 2009, 2010; Friston et al., 2023; Hipólito et al., 2021; Parr et al., 2022; Ramstead et al., 2021) were further developed. A common theme across these theories is Prediction Error Minimization (PEM). We can explain perception, learning or action as minimizing the prediction error (or its upper bound, free energy) in regards to the external environment.

The EMH is important for FEP and predictive processing theories. EMH supports the idea that some objects are part of our cognition and that the boundaries of cognition are not fixed. From the FEP perspective, this matter needs an answer, as those special objects (like phones, computers, or other people) play a vital role in the reduction of free energy compared to normal objects (like a rock or a river). Human agents can learn more about the weather from the weather app on their phone than from other objects with no special properties. The answer can take two directions: one in which we support EMH and accept that the mind is extended, and one in which we deny this. Accepting that EMH is true has the advantage of explaining how these special objects have such a role in the reduction of free energy (Beni, 2021): because they are part of our mind (Clark, 2016, 2025). However, it creates issues within the FEP framework itself, as we will see. The second answer is to deny that EMH is correct and to place a clear evidentiary boundary between the agent and the external environment (Hohwy, 2016). This version preserves the integrity of the FEP framework, but needs to find an answer to the question of why some objects have a disproportionate role in the reduction of free energy. Some objects are playing more than the role of being objects of our statistical processing; they are themselves doing some of the processing, or at least greatly helping our own. I can look outside and try to understand if it will rain in the next 2 h, or I can check the weather app and get the answer from there. The intuition is that there is a difference between the clouds and the phone. The clouds are part of the sensory input upon which I can build an internal model for prediction, but the weather app seems to provide an answer that greatly improves predictive power.

The aim of this paper is to understand whether and how the explanatory role attributed by the EMH to some special objects can be accommodated without weakening the explanatory constraints provided by the standard FEP architecture. MBs provide a formally defined evidentiary boundary between internal and external states, and allowing this boundary to shift so as to incorporate external resources may reduce its usefulness as a principled way of specifying the system under investigation. Importantly, rejecting an extended interpretation does not leave the FEP without resources for explaining the distinctive role of other agents and external objects. Existing accounts appeal to reciprocal inference (Friston K. J. and Frith, 2015), generative-model alignment (Friston K. and Frith, 2015), and socially structured active inference (Apps and Tsakiris, 2014; Veissière et al., 2020). Building on these FEP-compatible approaches, I propose Direct Input Variables (DIVs) as a more specific account of how the outputs generated by external systems can contribute to an agent's own inference without becoming constitutive parts of that agent's mind. The solution builds on the hypothesis that free energy can be decreased not just by building statistical models, but also by directly using as inputs the outputs produced by humans, computers, or other special objects, without performing the statistical inference itself, that is, by using DIVs. Reducing free energy can be achieved either by statistical inference performed by the brain or by using DIVs. The use of DIVs removes the need to accept EMH and preserves the integrity of the FEP framework, but it opens new questions regarding how DIVs can be integrated within the FEP framework.

## 2 The EMH and predictive processing theories

Because some cognitive processes that can be performed in the brain can also be carried out using external objects, or can be significantly improved by their use, it follows, according to the argument of EMH, that the mind is extended and that not all cognitive processes occur within the skull (Clark and Chalmers, 1998; Hutchins, 1995; Wilson, 1994). This is different from the internalist view that the brain is all it takes to explain all cognitive processes and the mind. The claim of EMH is that external objects are metaphysically constitutive parts of the mind (Kirchhoff and Kiverstein, 2019) and this is the version of EMH that I am going to discuss in this paper.

Predictive processing theories face a dilemma when addressing EMH: on the one hand, nobody can deny that certain external objects pose an important role in minimizing prediction error, and one solution is to accept that cognition and the mind are extended to certain external objects. On the other hand, accepting that the mind is extended seems to go against the carefully built framework of predictive processing. Taking the FEP as an example, MBs are a key component of the framework. An MB defines a boundary between a system and everything else, in our case between the brain and the external world, and allows for the building of statistical models of the causal relationships present in the external environment without the need for direct access to it (Friston, 2010, 2013; Kirchhoff et al., 2018; Kirchhoff and Kiverstein, 2021). If the brain has a MB, then it appears that this will generate a separation line between the brain and the external world; thus, all that is outside the brain will not constitute the mind. However, another option is to move in the direction of a permeable MB that does not have a fixed boundary, which would allow for the acceptance of EMH. My approach is to:

1. Start with the FEP as the guiding framework.
2. Accept the intuition of EMH as being correct: some objects are special when it comes to PEM, and this fact requires an explanation.
3. Deny EMH in order to preserve the integrity and simplicity of the FEP framework.
4. Propose a different explanation for the intuition of EMH by introducing the Direct Input Variables (DIVs).
5. Explain how DIVs constitutes a better solution by accounting for the intuition underlying the EMH, without the need to accept the EMH or change the FEP framework in any major way.
6. Discuss the counterarguments and the potential limitations of the DIV approach.

## 3 The FEP framework

The FEP claims, in its maximal form, that we can explain everything related to the brain and mind by the imperative of reducing free energy (Friston, 2009, 2010). Free energy is a measure of the uncertainty of an organism, including human agents, in relation to its environment. Perception, learning, and action can all be understood as facets of the same imperative of reducing uncertainty in relation to the external environment (Friston et al., 2010; Parr et al., 2022; Smith et al., 2022; Stubbs and Friston, 2024). The FEP comes with a robust mathematical apparatus (Bogacz, 2017; Buckley et al., 2017), which brings precision and structure to the general framework by showing how an agent can reduce the prediction error by minimizing free energy. Free energy has a bound on prediction error; hence, minimizing free energy will result in the PEM as well. I am going to use free energy minimization and PEM interchangeably in this paper. By defining free energy as a measure of the uncertainty of the agent in relation to its environment, we can accomplish the imperative of reducing free energy in two main ways: we can either develop better models to explain upcoming sensory input, hence improving prediction and reducing uncertainty, or we can directly change what we perceive so that we perceive what we expect based on our internal models (Friston et al., 2016, 2017; Proietti et al., 2025). There is great flexibility when it comes to reducing free energy, with the allocated precision of internal models being an important factor when deciding what is the best course of action in the aim of reducing free energy (Clark, 2013, 2018; Limanowski et al., 2024). High precision of the internal model will place the emphasis on action, changing perception so that the upcoming sensory input matches the models. If high precision is assigned to the internal models, we may render contradicting sensory input as having low importance (Lawson et al., 2014; Proietti et al., 2025). If I know for sure that I put the keys in the drawer, I will continue to look for them even if, at first glance, they are not there. But if I have no idea where they might be, only that it is probably in the house (low precision of my model), I will not insist when I do not see them in the drawer.

The brain can build statistical models of the external environment by using sensory input generated by hidden causes (Friston et al., 2012). By acting in the environment, the models are confirmed or a prediction error is generated. In this never-ending cycle, the MB allows the brain to build statistical models of the hidden causes, while they remain hidden. MBs can be understood as instruments for modeling how the brain works, by providing the mathematical structure to model the brain's interaction with the external environment in a statistical way (Bruineberg et al., 2022; Kirchhoff et al., 2018). But MBs can also be understood as actual real objects in the form of membranes, sensory systems, or the nervous system (Friston, 2013; Parr et al., 2019).

## 4 The intuition brought by EMH is correct

A common theme runs through the opinions of those who support EMH under the PP framework: the active use of external objects leads to a reduction of prediction error and free energy. I am going to discuss some of the approaches that support the alignment of PP or FEP with EMH, with the declared aim of agreeing with the intuition that some objects are special when it comes to PEM, and with the imperative to find a way to explain how this is possible. Supporters of EMH rely on the peculiar role of some objects and take the route of accepting them as part of our cognition. My view is to accept their intuition but propose a different solution, while denying that mind is extended.

Constant et al. argue that cognitive processes are not limited to the brain functions because organisms actively construct cognitive niches that function as parts of their cognitive system. Cognitive niches are environmental resources like tools, traces, cultural practices etc. that store information and guide action via affordances. The organism and environment form a coupled system jointly minimizing uncertainty, effectively treating the environment as part of the cognitive machinery (Constant et al., 2022). In this way, cognition is extended beyond the skull not just conceptually but as a shared, distributed process across brain, body, and world, thereby providing a formal justification and expansion of the extended mind hypothesis. Cognitive processes are not restricted to the brain processes but are sometimes constituted by dynamic interactions across the brain, body, and environment. External factors are not mere causal inputs, and at the base of perception we find ongoing embodied engagement and “extended dynamic singularities,” where internal and external processes form a single cognitive system with flexible, shifting boundaries. Cultural practices, tools, and social interactions can thus be part of the constitutive basis of cognition (and of consciousness) in certain situations (Kirchhoff and Kiverstein, 2019). Cognition is action oriented arising from a mechanistic system comprising brain, body, and environment with the aim of supporting PEM (Venter, 2021).

Even if we start with a purely brain-centric view of predictive processing, we can observe that cognition depends on embodied action, affective regulation, and continuous interaction with environmental structures, including social and cultural scaffolding. Human agents naturally recruit external resources like tools, cultural practices, environments which could be interpreted as cognition functionally spread across brain–body–world systems (Nave et al., 2020). It seems that the logic of predictive processing strongly motivates and explains how cognitive processes can extend into the environment, especially through epistemic actions and environmental scaffolding, thus positioning the extended mind as a natural consequence of the PP framework. Some have gone even further and saw the way we use LLMs as natural development accounted for by the EMH (Clark, 2025).

## 5 MBs and the case against EMH

There are three main ways in which we can approach the use of MBs in order to decide whether the mind can extend or not. One way is to deny that the mind is extended by considering that MBs define an evidentiary boundary that separates the brain from the external world. I will take this route. A second approach would be to allow for permeable MBs. A third approach is to deny that MBs have any value in deciding whether the mind is extended or not.

The use of MBs can help us when explaining how exactly the brain minimizes free energy. The view is that the brain has internal models that can be influenced only by sensory states, while being able to influence only active states. In this view, the external environment remains hidden, and the brain does not have any direct access to it (Kirchhoff, 2018b). The external environment produces the sensory input that is then compared with internal predictions, resulting in a confirmation of the model or in an error of prediction. Human agents can, in turn, act on the environment via active states to confirm their internal models or solve the resulting prediction error. Free energy is the difference between the generative model and the recognition model of the brain. The generative model is a way of explaining sensory input by having an internal model of how these inputs are generated by hidden causes. It is a model of the causal relations of external objects as they generate sensory input in the way we receive it. If the model is correct, the predicted sensory input will match the actual sensory input. The recognition model is a statistical model of the causal relations of hidden causes. It can be understood as the working model that is encapsulated in the brain, and it concerns how the brain believes the world is organized. It approximates the posterior distribution over hidden states given sensory input. What the brain does, according to FEP, is to minimize the difference between these two models, in such a way that its view of the world becomes as close as possible to the sensory input it receives. As we can see clearly from Figure 1, using the MB we can model the causal relationships within the external world without any direct access, but by using the indirect generation of sensory input and action on the external environment so that new input is generated to match internal models. From Figure 1, we can see that if we accept that the mind is extended beyond the evidentiary boundary and includes external objects, then the whole simplified picture of how the brain works will collapse. Assuming that the mind is extended to the phone, for example, would imply that the phone is part of the internal models, while also being part of the hidden causes that generate sensory input and are acted upon (I can see the phone and take it in my hand). The evidentiary boundary would be permeable, and the whole system would have to be replaced with something else. It does not seem that an object can be both inside and outside the evidentiary boundary while still preserving the whole FEP framework. Consider a hidden cause *h*_*i*_, in this case your phone. If *h*_*i*_ is inside the MB, then it should be a sensory state, an active state, or an internal model. It is not connected to our sensory system or to our motor system, so it must be part of our internal models, which is also in line with EMH. However, being part of the internal models means that it can be influenced only by sensory states and can influence only active states, while keeping hidden causes out of this process. But it is not at all clear how we can support the idea that the phone is influenced in any way by sensory states. Moreover, I can see the phone; hence, the phone is clearly acting as a hidden cause that generates sensory input. The phone can be *acted upon* which makes it clearly a hidden cause that can be acted upon to generate new sensory input. While this critique may seem simplistic, I believe that we should find a different solution to explain how the phone can contribute so much to PEM, rather than accepting the phone as part of the brain's MB and confusing the whole framework.

**Figure 1:**
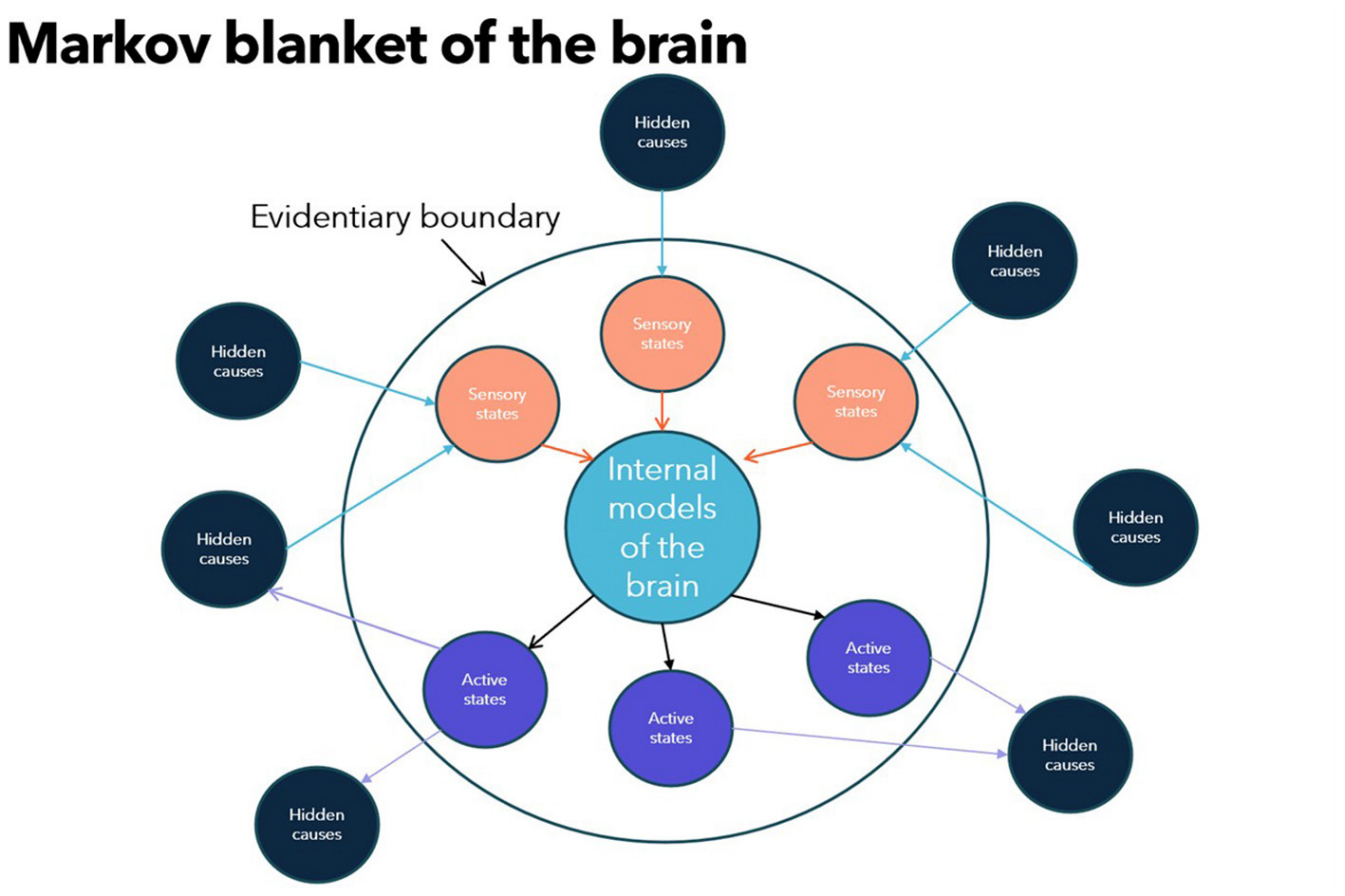
Markov blanket of the brain (drawn by the author).

While my claim is that we should reject EMH in order to preserve the integrity of the FEP, not all authors have the same opinion. One idea pursued is to argue that MBs do not define a fixed, brain-boundary of the mind but instead specify boundaries that are dynamic, nested, and potentially involving other objects from the external environment (Allen and Friston, 2018; Clark, 2017; Constant et al., 2022; Kirchhoff, 2018a; Kirchhoff et al., 2018). This is in line with the idea that the mind can extend beyond the brain and even beyond the body. While the MB formally partitions internal, sensory, and active states, the authors show that there is no single privileged blanket: boundaries can shift over time and operate at multiple scales, sometimes incorporating environmental elements when they participate in the system' self-evidencing dynamics. Because the boundary of the cognitive system is determined by processes that maintain the agent's organization through active inference, external resources, like phones or computers, can become part of the MB when they contribute to minimizing uncertainty and sustaining the agent's functioning. This directly supports the extended mind hypothesis by reframing cognition as a self-organizing system whose boundaries are fluid and can expand into the environment, rather than being fixed at the skin or skull (Kirchhoff and Kiverstein, 2021). Because cognitive systems are constituted by multi-scale, diachronic processes that recruit both internal and external resources, the MB becomes a flexible, process-defined boundary that can temporarily include elements of the environment when they participate in the system's PEM (White et al., 2025). We are talking here about coupled processes spanning brain, body, and environment, with no single privileged boundary—what counts as the “cognitive system” depends on the scale and explanatory context, making cognition a distributed, self-organizing, and context-dependent phenomenon (Ramstead et al., 2021). Cognitive boundaries should be determined by participation in PEM, not by location (Kersten, 2022).

- sensory states can only be influenced by hidden states and influence internal states (models),
- active states can only be influenced by internal states,
- internal states can only be influenced by sensory states and influence active states,
- hidden causes have no direct contact with the internal states. They can only influence sensory states and be influenced by active states (Ramstead et al., 2023).

Considering these two conflicting approaches, that MBs have a clear demarcation line or that MBs are permeable and have shifting boundaries, another approach is to deny that we can use MBs to decide on the question of weather mind is extended or not. Facchin argues that MBs cannot be used to determine whether cognition is confined to the brain or extends into the body and environment, because their application is fundamentally circular, model-dependent, and underdetermined: one must already identify the system of interest before defining its MB, and since blankets are multiple, nested, and often selected based on explanatory goals, they fail to provide an objective or principled boundary of the mind (Facchin, 2023).

In the same direction, Menary and Gillett argue that MBs fail to demarcate the boundaries of the mind, since any successful use of MBs for this purpose should reflect a real division in nature. Under Pearl's account, MBs are purely statistical constructs in a Bayesian network, representing the minimal set of variables that render a node conditionally independent from the rest of the network, thus functioning as a tool for efficient inference without making ontological claims about real boundaries (Pearl, 2009, 2014; Shpitser and Pearl, 2006). Other interpretations support MBs as being real objects, like membranes in a cell (Kirchhoff et al., 2018; Ramstead et al., 2020; Wiese and Friston, 2021) but according to Menary and Gillett, these approaches are making an unwarranted jump from mathematical formalism to a metaphysical claim (Menary and Gillett, 2022) even if we consider that steps have been made in this direction (Friston et al., 2023). The matter of EMH should be decided by empirical investigation (de Bruin and Michael, 2017) which would take the debate to favor the realism of MBs.

## 6 Introducing direct input variables

Regardless of the path chosen when discussing the problem of MBs as a useful tool for deciding the truth of EMH, there is widespread agreement that objects like phones, computers or other human agents play a disproportionate role in PEM. These objects can help PEM much more than a rock and an explanation for this fact needs to be provided. Accepting the view that MBs are flexible and extendable is obviously a good explanation for this fact, but it strikes at the heart of the FEP framework. Denying EMH based on the clear evidentiary line imposed by the brain's MB creates the need to explain the fact that external objects have a disproportionate role in PEM. As I am advocating for this path, I will need to explain how FEP and PP theories can account for this fact while denying EMH and preserving the framework. The third path—to deny that MBs are a useful tool in deciding whether EMH is true or not—keeps the issue of explaining the role of some special objects in PEM on the table and, I believe, essentially denies FEP.

I am proposing a different path by accepting that MBs provide a clear evidentiary boundary, preserving the FEP, while introducing Direct Input Variables. While most of the time we perform statistical inferences ourselves, sometimes we use other objects for this purpose. Consider the question of whether to take an umbrella when I am going for a walk. In one instance, I can look outside and check whether I can see raindrops. I move toward the window, look outside, and recognize the rain or the lack of it based on my internal models. The output of these actions is clear in this case: “it is raining outside.” I have compiled this output based on statistical inference performed by the brain. I can then use this output as input for another inference—whether to take the umbrella—and I decide to take it. This is very much in line with the FEP story.

Now consider a different situation. I have the same question about whether I should take an umbrella and need the same input—whether it is raining or not. The difference this time is that instead of performing the statistical inference myself and producing the output “it is raining,” I can do something different: I can ask my wife to check it for me. She will perform a similar statistical inference and conclude that “it is raining.” She will pass this information to me, and I will then use her output as input to my own model when deciding whether to take the umbrella. I call the output provided by my wife a Direct Input Variable, because I can use it as input without needing to perform the entire statistical inference myself. Of course, I can assign a probability to this input, but this is also true of my own statistical inference processes.

The intuition behind introducing DIV is based on the hierarchical nature of our brain, which can be understood as MBs within MBs (Friston et al., 2021; Hipólito et al., 2021; Kirchhoff, 2018a; Kirchhoff et al., 2018), where lower levels' outputs represent the inputs of higher levels. The brain can be understood as a hierarchy of nested MBs (Ramstead et al., 2023), where statistical boundaries partition neural systems into interacting units across multiple scales—from neurons to microcircuits, brain regions, and whole networks. At each level, internal states are conditionally separated from external states by sensory and active “blanket” states. This structure is recursively applied, meaning that each component is itself a MB system embedded within larger ones, forming MBs of MBs. The interpretation is that our brain is accustomed to using the outputs of some models as inputs to other models. My conjecture is that we are simply extending this capability to external systems, such as other humans.

There are some conditions that need to apply in order for this to be possible: first, the DIV should come in a form that can be inputted into the internal model; and second, the source of DIV needs to have enough precision assigned so that it can be used in other internal models.

The first condition ensures that the DIV comes in a form that can be incorporated into the brain's own models. If you talk to me in Japanese and I am not a Japanese speaker, I cannot use the DIV. The agent does not need to be able to generate this output independently, or even to verify it. What matters is that the agent can use it in a subsequent inference. I might not be able to crack a cryptographic code, but this does not preclude me from using the result produced by a computer that deciphers that code. The first condition therefore does not require that the brain be capable of performing the same statistical inference as the external system. An agent may use the result of a highly complex computation produced by a computer despite being unable to reproduce or independently verify that computation. What is required is that the agent understands how the resulting variable can be used in a subsequent inference.

The second condition involves assigning a probability to DIVs that makes them usable in the brain's internal models, considering the fact that low-precision inputs are disregarded by the brain.

Both conditions ensure that DIVs work in a similar way to internal inputs. This similarity, along with the hierarchical nature of the brain, is what allows for the use of variables both as outputs of statistical inference but also as input for another statistical inference.

## 7 Discussion

By introducing DIV, I propose a way of accepting FEP while denying EMH. The advantage of this approach is that it preserves the FEP while accounting for the intuition underlying the EMH—namely, how certain objects contribute significantly to PEM. All objects that fall under EMH fall under DIV, but the reverse is not true. Many objects with which we do not have a reliable connection can serve as DIVs, even objects we encounter for the first time or to which we assign a sufficiently high probability such that we can use them as DIVs.

The first condition is that the input comes in a form that can be incorporated into existing models. Although this is not explicitly discussed in EMH, it is consistent with it. In the well-known case of Otto's notebook, he is the one who actually made the notes, hence he can re-use it as input. The second condition is what makes the difference: we can accept many types of input as DIVs, and they do not necessarily need to be reliable, easily accessible, or meet other conditions typically invoked to strengthen the EMH claim. We use the input as a DIV depending on the assigned probability, which in turn depends on many factors considered within our own modeling of the external world. DIV is therefore much more flexible than EMH, without requiring the disputable claim that external objects are part of our mind.

This is different from other explanations that were provided. According to Friston, communication between two people is a process of reciprocal mutual inference in which each person continuously tries to predict the other's behavior and the sensory signals they generate. Rather than transmitting fixed “information,” each agent uses a shared or similar internal model to interpret incoming signals as evidence about hidden causes like intentions and meanings, updating their beliefs by minimizing prediction error. At the same time, they act to make their own signals predictable to the other. This creates a loop of alternating listening, which can be understood as making predictions, with speaking, which can be understood as acting in the world. This is modulated by attention and precision weighting, which lead to gradual alignment of the internal states of the two communicators. When successful, this process leads to a temporary synchronization of their internal models, enabling them to effectively “share a narrative” and understand each other (Friston K. and Frith, 2015). This is very much in line with the traditional FEP story. By introducing DIV, I am supporting a different view, namely, that we are actually transmitting “information” in a much more direct way. We are still applying probability to this “information,” but without the full extent of the usual statistical processing. Shared neural and behavioral patterns are understood as important for successful communication (Hasson and Frith, 2016), but what I am adding is that the shared neural patterns are just the instantiation of being prepared to receive the output of other people's statistical modeling. The process of inferring other agents' expectations about the world and how to behave in a social context, dubbed as “thinking through other minds” (Veissière et al., 2020), can also be understood under the DIV proposal as aligning internal readiness to receive the outputs of other objects that perform statistical inferences as inputs to our own models.

I am not denying that communication between two people facilitates long-term changes in generative models (Friston K. J. and Frith, 2015) and that there is a dual prediction and modeling of the other in such a way that when we communicate successfully, an alignment takes place between the two or more communicators. Each person's brain builds internal models (priors) of others' intentions, meanings, and likely actions, using past social experience and shared cultural “typifications” (e.g., norms, roles, language patterns), and then updates these models by minimizing prediction errors during interaction. Communication is therefore not just the transmission of information but an active inference loop, where people both interpret signals (speech, gesture, context) and act (speak, gesture, adjust behavior) to make others more predictable and to reduce uncertainty. Successful communication occurs when these internal models become sufficiently aligned—through shared expectations, intersubjectivity, and ongoing feedback—allowing smooth coordination of meaning, while misunderstandings arise when prediction errors persist or models diverge (Kelly et al., 2019). I agree with these views, and I am taking a step forward and being more precise about how this is happening, namely by using the outputs of other's models as inputs to our own internal models. This is not against FEP. While I accept that modeling others and mirror neurons play a role (Kilner et al., 2007), I am adding something extra that is not part of the FEP story in an obvious way.

Modeling others can be linked with self-modeling. Communication with others relies on self-awareness processes, such as recognizing one's own voice or body, which enable individuals to distinguish themselves from others and interact effectively. It emerges through the brain's integration of sensory signals and predictions, allowing people to interpret and respond to others by aligning internal expectations with incoming social information (Apps and Tsakiris, 2014). The source of us being able to accept the output of other people as input variables to our own internal models stems from the extension of our ability to use outputs of internal models as inputs to other internal models. What DIV adds in this discussion is the fact that it points to how this is possible and, more importantly, what are we predicting when interacting with others. We are not just predicting others, but we are also using their outputs as direct inputs to our own statistical models. People align with each other through verbal and non-verbal signals, using feedback, mimicry, and context to build common ground and iteratively adjust their interpretations (Kahl, 2020; Kahl and Kopp, 2018; Tamir and Thornton, 2018), but they are also simply exchanging direct input variables, and all the alignment with others may be aimed at increasing the precision of this exchange.

None of the above leads in any way to the need to accept EMH. We can still preserve the evidentiary boundary of the brain's MB and explain why interacting with some external objects leads to PEM. This is because we can use their outputs as direct input variables into our statistical models, leading to better predictions. There is nothing that leads to the need to accept EMH. We still have a brain-centric view, predicting and modeling other people in full alignment with the predictive processing view. But we have learned that others can offer direct input variables, and we assign a high probability to some of these variables, using them as inputs to our internal models.

If accepting DIVs can be a solution to the problems raised by the EMH for the FEP, this can also potentially undermine the FEP. Hemmatian et al. argue that the FEP is unlikely to serve as a genuinely unifying theory of brain function because it cannot fully explain human decision-making without introducing the notion of subjective utility, which is independent of the FEP (Hemmatian et al., 2024). The authors therefore propose treating FEP-related informational objectives and utility as distinct but interacting determinants of behavior, formalized in a two-factor model whose relative weights vary by context. On this view, prediction-error and uncertainty reduction matter primarily when they contribute to an agent's goals, rather than constituting universal motivations in themselves. The FEP may thus remain highly useful within specific domains, particularly where information optimization and utility align, but should be integrated with reinforcement learning, decision theory, and independent models of subjective preference rather than treated as an all-encompassing principle of cognition. The question arises whether DIVs represent a similar potential threat to the FEP. At first glance, this does not seem to be the case. Learning the reliability of an information source can be modeled within hierarchical Bayesian inference, including through higher-order estimates of the stability or volatility of that source over time (Diaconescu et al., 2014). Thus, at least when it comes to the intentions of others, we do not need to move beyond existing Bayesian mechanisms to explain why an externally supplied variable can be assigned more or less precision depending on the inferred reliability of its source, without requiring the source itself to become part of the agent's cognitive system. Discussing again the example of deciding whether to take an umbrella when going outside, while I can use a weather app to make this decision, I can also do it on my own by looking outside to check whether it is raining or whether clouds are gathering. The result of this statistical inference might be that there is a high probability of rain. This result is a variable that feeds into other calculations when deciding whether to actually take the umbrella. Thus, the brain already has a mechanism for using the results of statistical inferences as input variables for new statistical inferences. DIVs can therefore be understood as the way we treat an external variation of the same internal process. Understood in this way, the DIV proposal aligns well with the FEP, but it does not solve the problem raised by Hemmatian et al.

## 8 Conclusion

Accepting the DIV approach may allow us to explain the intuitions of EMH without weakening the standard FEP framework. Some objects have a disproportionate contribution to PEM not because the mind is extended to these objects, but because human agents can use their outputs as DIVs. This is very much in line with FEP and predictive processing in general, and it has the advantage of capturing the intuition of EMH, without accepting the EMH. The DIV view leans toward an internalist perspective and explains the apparent porous nature of MBs as a special use of the same statistical modeling performed by the internal apparatus, without the need to change the nature of MBs' evidentiary boundary. This is very much in line with how predictive processing and FEP treat social interaction, and it suggests that social networks may have emerged in the first place as a way of decreasing free energy by using DIVs from other human agents. A more detailed explanation of how the brain accommodates DIVs should be provided.

DIVs can therefore explain the distinctive epistemic role of other agents and artifacts while preserving a fixed evidentiary boundary, since the precision assigned to external sources can be learned in a Bayesian manner, in much the same way as the precision of other inputs. A separate solution needs to be provided to the problem of how agents select and use DIVs in relation to their goals, a question that connects the proposal to broader debates about whether preferences can themselves be derived from free-energy minimization.

## Data Availability

The original contributions presented in the study are included in the article/supplementary material, further inquiries can be directed to the corresponding author.
